# Allied health – 3008. Comparison of anti-allergic effect of pneumococcal conjugated vaccine and pneumococcal polysaccharide vaccine in a murine model of house dust mite allergic rhinitis

**DOI:** 10.1186/1939-4551-6-S1-P184

**Published:** 2013-04-23

**Authors:** Soo Whan Kim, Boo-Young Kim

**Affiliations:** 1 The Catholic University of Korea, South Korea

## Background

It was discovered that pneumococcal vaccine can reduce the allergic disease.The purpose of the present study is to evaluate the anti-allergic effect of pneumococcal conjugated vaccine and pneumococcal polysaccharide vaccine in a murine model of house dust mite allergic rhinitis. We also evaluated difference between effect of both vaccines.

## Methods

Forty mice were divided into four groups: control, Der f, pneumococcal polysaccharide vaccine (PV) , and protein conjugate polysaccharide vaccine (PCV). Allergic rhinitis was induced in BALB/c mice by intraperitoneal sensitization and intranasal challenge with *Dermatophagoides farinae* (Der f). The allergic symptom after the final challenge was recorded. Interferon (IFN)-γ, Interleukin (IL)-13, and IL-10 levels in nasal lavage fluid (NALF), as well as serum Der f-specific IgE levels were measured. The number of eosinophils in lamina propria was evaluated. The levels of T-bet, GATA-3, and Foxp3 mRNA expression in splenic mononuclear cells were determined by real-time polymerase chain reaction. A comparison of the frequency of CD4^+^CD25^+^Foxp3^+^regulatory T cells in splenic mononuclear cells were made by flow cytometry.

## Results

The T-bet m RNA level was lower in the PV and PCV group than Der f group (p< 0.05). The IL-13, GATA-3 mRNA level and serum Der f-specific IgE and eosinophil were lower in the PV and PCV group than Der f group (p< 0.05). Foxp3 mRNA expression in the PV and PCV group was elevated compared to the Der f group (p< 0.05). In flow cytometry, the PV group (p < 0.05) and the PCV group (p < 0.05) had higher percentages of CD4+ CD25+Foxp3+ T cells than the Der f group. In the PV group, the percentage of these cells was higher than that in the PCV group. (p=0.00).

## Conclusions

These results suggest that the pneumococcal polysaccharide vaccines will suppress the allergen-specific Th2 response and enhanced the induction of regulatory T cells in a model of allergic rhinitis. And the process to work regulatory T cell can be different between the PV and PCV group.

